# A new species of *Agaricus* section *Minores* from China

**DOI:** 10.1080/21501203.2015.1121931

**Published:** 2015-12-16

**Authors:** He Mao-Qiang, Zhao Rui-Lin

**Affiliations:** a Key Laboratory of Forest Disaster Warning and Control Yunnan Province, Forestry College, Southwest Forestry University, Kunming650224, China; b State Key Laboratory of Mycology, Institute of Microbiology, Chinese Academy of Sciences, Beijing100101, China

**Keywords:** Agaricaceae, phylogeny, morphology, taxonomy, ITS

## Abstract

*Agaricus gemloides* sp. nov. is characterised by its reddish brown fibrillose squamose on the pileus, relatively slender basidiome and broader basidiospores. In this article, it is introduced based on its distinguished morphological features and molecular phylogenetic position.

## Introduction

*Agaricus* L. (Agaricaceae, Agaricales) is a genus more than 400 species right now (Zhao et al. 2011, 2012; Chen et al. 2012, 2015; Lebel & Symel 2012; Lebel 2013; Li et al. 2014; Gui et al. 2015; Liu and Hyde 2015; Wang et al. 2015). It is also a valuable genus and contains a lot of important species, such as the famous cultivated mushrooms *A. bisporus* (J. E. Lange) Imbach and *A. subrufescens* Peck. A taxonomic system with eight sections of this genus was established mainly based on samples from Europe and North America (Cappelli 1984; Kerrigan 1986; Parra 2008, 2013). Recently, phylogenetic analysis on *Agaricus* from tropical areas revealed 11 new lineages (Zhao et al. 2011). The deeply phylogenetic studies on those new lineages indicated that several sections had been reconstructed or recognised, such as sections *Brunneopicti*, and *Nigrobrunneoscentes* (Chen et al. 2015; Wang et al. 2015).

The word *Minores* was firstly proposed in 1874 by FRIES to name a group in *Agaricus* (Fries 1874). In 1952, Minoers was treated as a subsection under section *Arvenses* by Konrad et al. Until now, it was promoted in section level by the recent studies. And this section is well supported as a monophyletic group in all those studies (Nauta 2001; Zhao et al. 2011; Chen et al. 2012; Lebel 2013; Parra 2013; Liu and Hyde 2015). Species of *Minores* have a strongly positive yellow KOH reaction and positive orange Schäffer’s reaction on the surface of basidiome and context; annulus simple; anise or almonds smell. Twenty species of the section *Minores* in Europe were reported (Parra 2013), and many new species of *Minores* were also reported in recent years from worldwide, such as *A. megalosporus* J. Chen, R. L. Zhao, Karunarahtna & K. D. Hyde, *A. sodalis* L. J. Chen, R. L. Zhao & K. D. Hyde and *A. parvibicolor* L. J. Chen, R. L. Zhao & K. D. Hyde from Thailand (Chen et al. 2012; Liu and Hyde 2015); *A. lamelliperditus* T. Lebal & M. D. Barrett and *A. colpeteii* T. Lebel from Australia (Lebel 2013). A recent mushrooms survey from southwest China reveals several new species, *A. gemloides* is introduced in this article.

## Materials & methods

### Morphological study

Every specimen was photographed in the field, wrapped in aluminium foil and kept separately in plastic box. Macro features of fruitbody were recorded (including the features of cap, lamella, stipe, annulus and the size, colour, odour of the fruitbody) when back room. Then specimens were dried completely in a food drier at 70°C kept in plastic bags, and deposited in Herbarium Mycologicum Academiae Sinicae (HMAS), Beijing. Herbarium acronyms are those of Thiers (http://sweetgum.nybg.org/ih/ continuously updated). Micro features were recorded under microscope (Olympus CX31) following the method described by Largent (1986). At least 20 measurements were made for each features (including basidiospores, basidia, cystidia, pileipellis and annulus hyphae). Data were recorded as follows: *X* = the mean of length by width ± standard deviation (SD); *Q* = the quotient of basidiospore length to width and *Q*_m_ = the mean of *Q* values ± SD.

### Phylogenetic study

DNA was extracted from the dried fruiting bodies using the E.Z.N.A. Forensic DNA Extraction Kit (D3591-01, Omega Bio-Tek). Following the protocol of White et al. (1990), the internal transcribed spacer (ITS) regions were amplified. Sequencing was performed in commercial biotechnology company (Biomed). Newly generated sequences along with the reference sequences from GenBank were aligned using ClustalX 2.0 (Thopmson et al. 1997), and then adjusted manually in BioEdit v. 7. 0.4 (Hall 2007). The alignment has been submitted to TreeBase (submission ID 18397). Bayesian analysis was performed with MrBayes 3.1.2 (Ronquist et al. 2003). GTR+I + G (GTR: General time reversible; I: proportion of invariable sites; G: shape parameter of the gamma distribution) was chosen as the best model for nucleotide substitution by MrModeltest 2.2 (Nylander 2004). Run one million generation for six Markov chains, and sampled every 100th generation resulting in 10,000 trees. Those trees sampled prior to searches reaching a split deviation frequency value reaching 0.01 were discarded as the burn-in, and remaining trees were used to calculate Bayesian posterior probabilities of individual clades. Maximum likelihood was performed in PAUP*4.0b 10 (Swofford 2004), the best substitution model GTR+I + G was selected by hLRT in MrModeltest 2.2 (Nylander 2004). Maximum parsimony analysis was also performed using PAUP*4.0b 10 (Swofford 2004). One thousand heuristic searches were conducted with random sequence addition, tree bisection-reconnection (TBR) branch swapping and gaps treated as missing data. Parsimony bootstrap values were obtained from 1000 bootstrap replicates, with starting trees obtained via stepwise addition, random sequence addition, and max-trees set to 1,000,000.

## Results

### Phylogenetic analyses

**Figure 1.**
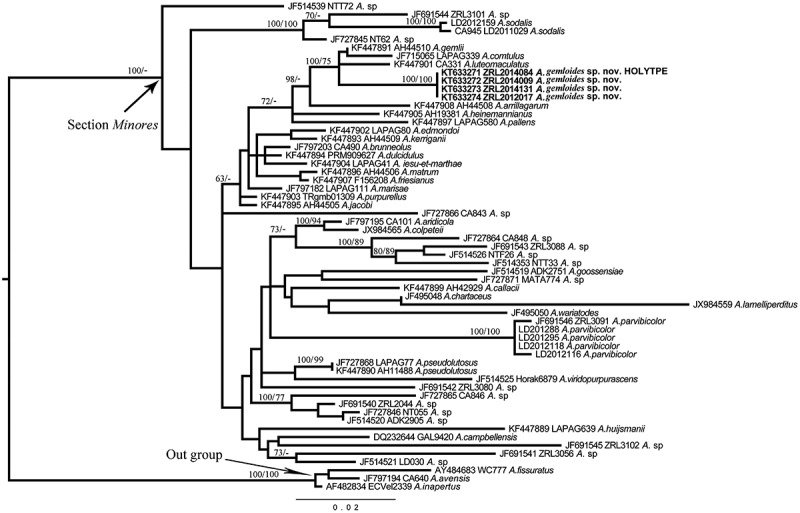
Phylogeny of *Agaricus* section *Minores* generated from maximum likelihood analysis of ITS sequences rooted with *Agaricus fissuratus, A. avensis and A. inapertus*. Bayesian posterior probability (PP) values above 60% and parsimony bootstrap support (BS) above 50% are given at the internodes (PP/BS).

The dataset contains 59 ITS sequences, of which four are newly generated from this study and the rest of the sequences are retrieved from the GenBank. In this dataset, 29 known species are included, *Agaricus arvensis* sensu Cooke, *A. fissuratus* F. H. Møller, and *A. inapertus* Vellinga were chosen as the outgroup (Zhao et al. 2011). Except for those three outgroup species of the section *Arvenses*, the rest species are all belonging to the section *Minores*. Totally, 702 characters containing gaps are rested in final alignment, among which 479 are constant, 140 are parsimony-informative, and 83 are parsimony-uninformative. The section *Minores* is monophyletic group, which is highly supported with high PP and BS values (100/-). Trees generated by MP, ML and Bayesian methods exhibit almost the identical topology. The proposed new species group together with *A. gemlii* L. A. Parra, Arrillaga, M.Á. Ribes & Callac (AH44510), *A. comtulus* Fr. (LAPAG339), *A. luteomaculatus* F.H. Møller (CA331). Differences are noted in the position of *A. viridopurpurascens* Heinem (Horak6879), it groups with this four species together only in ML tree. The ML tree is shown in Figure 1. Bayesian posterior probabilities (PP) and parsimony bootstrap support (BS) values are given at the nodes (PP/ BS). The proposed new species is represented by four specimens, zrl2014084, zrl2014009, 2014131, and 2012017, which are shown to be monophyletic with high support (100/100). The phylogenetic analyses support those four specimens as one distinguished species.

## Taxonomy

**Figure 2.**
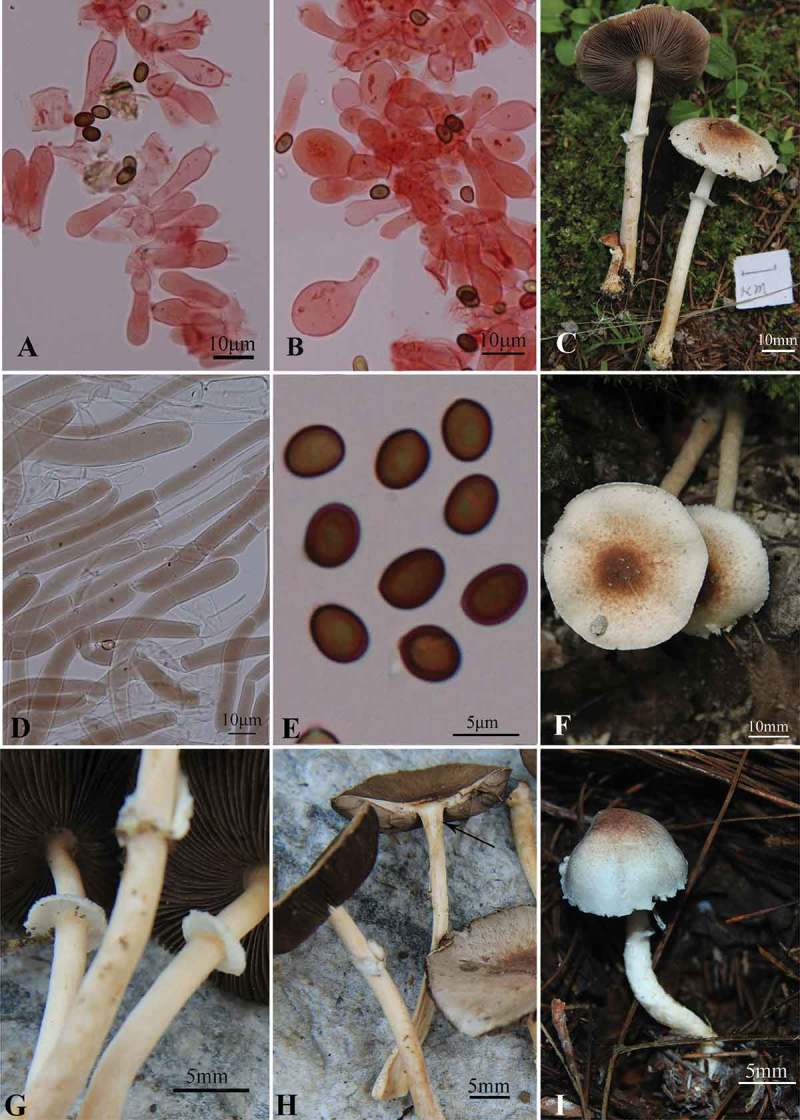
Morphology of *Agaricus gemloides*. A. Basidia B. Cheilocystidia; C. Basidiome when mature; D. Pileipellis hyphae; E. Basidiaspores; F. Pileus; G. Annulus; H. Context discolouring on cutting; I. Basidiome when young. (A–F from specimen zrl2014084 Holotype; G, H from specimen zrl2014131, and I from specimen zrl2012017).

*Agaricus gemloides* M. Q. He & R. L. Zhao sp. nov. (Figure 2).

Fungal Names: FN570215

Etymology: refers to its morphological similarity with *A. gemlii*.

Macroscopical characteristics: *Pileus* 14–36 mm in diam., parabolic when young, then convex and plane with age, margin slightly exceeding, straight, occasionally uplifted when mature, surface dry, covered with reddish-brown fibrils radially, denser on disc and more scattered towards the margin. Background white or light brown. *Context* 1–3 mm broad, fleshy, white or light brown. *Lamellae* free, crowded, firstly pink or pinkish-brown, then brown, finally black brown when mature, with more than 7 series of lamellae, 1–4 mm broad, edge serrulated, paler. *Stipe* 18–76 × 2–4 mm (apex), 3–7 mm (base), cylindrical, slightly bulbous, hollow, surface white, above the annulus smooth, and below the annulus fibrillose. *Annulus* simple, membranous, white, 4–6 mm broad, pendant or peronate, smooth on both sides. Basidiome stain yellow on touching. Context turn yellow on exposure. Odour of almonds.

Macrochemical reactions: KOH reaction positive yellow. Schäffer’s reaction positive orange.

Microscopical characteristics: *Basidiospores* 4.3–5.6 × 3.3–3.9 μm, [*X* = 4.7 ± 0.2 × 3.6 ± 0.1 μm, *Q* = 1.3–1.4, *Q*_m_ = 1.3 ± 0.1, n = 20], ellipsoid or broadly ellipsoid, brown, smooth, thick-walled, without germ pore. *Basidia* 13.8–19.4 × 5.1–6.6 μm, clavate to long clavate, smooth, hyaline, 4-spored. *Cheilocystidia* 11.6–29.8 × 7.3–13.9 μm, single, smooth, hyaline, yellowish in mass or light brown sometimes, always be ellipsoid to broadly ellipsoid, also can be globose and spheropedunculate, with separated base sometimes. *Pleurocystidia* absent. *Pileipellis* a cutis composed hyphae of 2.8–12.7 μm, cylindrical, hyaline, brown, smooth, constricted at the septa. Hyphae of annulus 3.2–6.9 μm, cylindrical to short cylindrical, hyaline, smooth, slightly constricted at the septa.

Habitat: solitary, scattered or gregarious on soil, in forest.

Specimens examined: China, Yunnan Province, Dali, Cangshan Mountain, 26, July 2014, Xu Meng-Lin, *zrl2014084* (HMAS, 274006, HOLOTYPE); Yunnan Province, Kunming, YeYahu, 30, June 2012, Zhao Rui-Lin, *zrl2012017* (HMAS, 274002); China, Yunnan Province, Dali, Cangshan Mountain, 27, July 2014, Su Sheng-Yu, *zrl2014009* (HMAS, 274005); China, Yunnan Province, Dali, Cangshan Mountain, Gantong temple, 27, July 2014, He Mao-Qiang, *zrl2014131* (HMAS, 274008).

## Discussion

The ITS sequences analyses indicate that *A. gemloides* belongs to section *Minores*. And the morphological characters (yellow discolouration when cutting, yellow KOH reaction, almond odour, simple annulus) also conform the features of section *Minores*. This new species is characterised by slender basidiome, reddish brown fibrillose squamules on the pileus, and relatively broader basidiospores. Three species are closely related to *A. gemloides* in our phylogenetic analysis. They are *A. luteomaculatus, A. comtulus* and *A. gemlii. A. luteomaculatus* differs from *A. gemloides* by its white to ochraceous-brown pileus and bigger basidiospores (average size of 6 × 4 μm in Parra 2013). *Agaricus comtulus* is the type species of the section *Minores*, which has white pileus generally, but in some cases it can be light ochre, pinkish, light reddish-brown or reddish-purple colour (Parra 2013). *A. gemloides* can be differentiated from this known species by more slender basidiome and wider basidiospores (the diameter of stipe in *A. comtulus* is 3–9 mm in apex, 4–14 mm in base; and *Q* = 1.24–1.6 in Parra 2013). The species most morphologically similar to *A. gemloides* is *A. gemlii*, because both of them present same characters on pileus, lamellae edge, and cheilocystidia in shape, colour and size. But *A. gemlii* has bigger and narrower basidiospores (*Q* = 1.28–1.77) than those of *A. gemloides. Agaricus dulcidulus* Schulzer is another species which is quite similar to *A. gemloides* in the field, however this known species has very small basidiospores (3.6–4.85 × 2.6–3.3 μm). Furthermore, the molecular analysis supports *A. gemloides* as a distinguished species which is clear different from *A. comtulus, A. dulcidulus, A. gemlii* and *A. luteomaculatus*.

## Disclosure statement

No potential conflict of interest was reported by the authors.
